# Customized Career Development Platform (CCDP) for clinical and translational researchers: A pragmatic cluster-randomized controlled trial

**DOI:** 10.1017/cts.2023.687

**Published:** 2023-11-22

**Authors:** Doris M. Rubio, Colleen Mayowski, Emma A. Meagher, Cecilia M. Patino, Maya S. Thakar, Julie L. Welch, Gretchen E. White

**Affiliations:** 1 Institute for Clinical Research Education, University of Pittsburgh Schools of the Health Sciences, Pittsburgh, PA, USA; 2 Perelman School of Medicine, University of Pennsylvania, Philadelphia, PA, USA; 3 Keck School of Medicine, University of Southern California, Los Angeles, CA, USA; 4 Indiana University School of Medicine, Indianapolis, IN, USA

**Keywords:** Career development, career planning, individualized development plan, career success, career goals and milestones, competencies, mentoring

## Abstract

**Introduction:**

Early-stage clinical and translational researchers who set and track career goals, milestones, and progress are successful in career development. We aimed to determine the effectiveness of the Customized Career Development Platform (CCDP), an online individual development plan (IDP), versus the traditional IDP template in improving research success and career satisfaction.

**Methods:**

We conducted a pragmatic cluster-randomized controlled trial of 340 scholars and trainees at 27 US academic healthcare institutions. The primary outcome was number of published manuscripts 24 months post-intervention. Secondary outcomes included the number of grant proposals submitted and funded, job satisfaction, and level of communication with mentors. An analysis of CCDP participants assessed proficiency level for the 14 Clinical and Translational Science Award (CTSA) competencies. Data were analyzed using intention-to-treat.

**Results:**

Participants were mostly female (60.3%) and Caucasian (67.2%); mean age was 34 years. Twenty-four months following the intervention, the CCDP versus traditional IDP groups showed a similar number of publications (9.4 vs 8.6), grants submitted (4.1 vs 4.4) and funded (1.3 vs 2.0), and job satisfaction score (3.6 vs 3.7). The CCDP group had higher odds of discussing communication (OR = 2.08) and leadership skills (OR = 2.62) and broadening their network (2.31) than the traditional IDP group. The CCDP arm reported improvements in 9 of the 14 CTSA competencies.

**Conclusion:**

The CCDP offers CTSA hubs an innovative alternative to traditional IDP tools. Future studies are needed to elucidate why the CCDP users did not fully appreciate or adopt the functionality of the online platform.

## Introduction

The National Institutes of Health (NIH) requires all NIH-funded trainees to complete an individual development plan (IDP). An IDP is a tool that enables trainees to strategically plan their career development by setting short- and long-term career goals in conjunction with their mentor(s). A few IDP tools are currently available, such as myIDP [1]. The American Association for the Advancement of Science developed this free online tool, modeled after the Federation of American Societies for Experimental Biology’s Individual Development Plan for Postdoctoral Fellows [2]. However, the myIDP tool has two significant shortcomings regarding the needs of clinical and translational science trainees. First, it focuses primarily on early-career exploration in basic sciences, limiting utility for trainees at different career stages or with different research interests. Second, myIDP is not interactive in that mentors and or administrators do not have access to their mentees’ myIDP.

The Clinical and Translational Science Award (CTSA), awarded to over 60 academic health centers, funds KL2 scholars who are junior faculty and TL1 pre-and post-doctoral trainees. Given that the focus of the KL2 scholars and TL1 trainees is on clinical and translational research, some institutions developed their own paper IDPs to best suit their needs. However, paper-based IDPs are not accessible in real time from any platform to update goals, track competencies, monitor timelines, or communicate electronically and interactively, nor do they facilitate sharing data with the mentoring team and training program leadership. The lack of a centralized online platform for the IDPs makes it difficult for CTSA hubs with KL2 and TL1 programs to track their scholars’ and trainees’ metrics of success. Data show that 50% of these CTSAs report only fair to good compliance with using IDPs [3]. In fact, most KL2 scholars report that the development of an IDP was not helpful in their career success [4]. Potential reasons for the lack of enthusiasm about the value of an IDP include the lack of a CTSA scholar-specific IDP template and standards on how to implement, as well as the static nature of current platforms (i.e., paper-based).

Shortcomings of existing paper-based IDPs suggest there is potential benefit in creating an online platform for an IDP tailored specifically to the needs of CTSA scholars and trainees. The University of Pittsburgh’s Clinical and Translational Science Institute (Pitt CTSI) created an online Customized Career Development Platform (CCDP) for their scholars and trainees and were approached by several CTSAs interested in adopting it. The Pitt CTSI submitted and was awarded a National Center for Advancing Translational Science (NCATS) administrative supplement to make the CCDP a standalone platform to be broadly disseminated across CTSA hubs, and an R21 was awarded to evaluate its effectiveness. An investigative team was assembled and included the University of Pittsburgh, University of Pennsylvania, University of Southern California, and Indiana University, as well as a design firm to enhance the platform and tailor it for clinical and translational trainees and scholars. Through usability testing of the CCDP across several institutions via focus groups, interviews, and surveys, we improved the CCDP’s functionality and added several key features, such as an automatically generated Gantt Chart (Supplemental Figure 1).

The CCDP is interactive and readily accessible and allows scholars and trainees to track and communicate their competencies, goals, and progress. This platform benefits multiple groups such as: scholars and trainees by improving the ease in updating their goals and milestones: program leadership by increasing the transparency; and for mentors and mentees by improving communication between them. Our platform is intuitive, interactive, and meant to facilitate mentor–mentee relationships; it is a one-stop shop for trainees and scholars, mentors, and program administrators to track scholars’ and trainees’ goals linked to competencies.

We tested the effectiveness of the CCDP versus traditional IDP using a pragmatic cluster-randomized controlled trial to determine whether the CCDP improves research success and career satisfaction for scholars and trainees. We hypothesized that strategically planning one’s goals and milestones as well as the increased transparency and ease of communication afforded by the CCDP would lead to greater research success after 24 months, as measured by the number of published manuscripts, higher number of grant proposals submitted, higher number of grants funded, higher job and career satisfaction, and improved communication between mentees and mentors.

## Methods

Participating CTSA hubs were randomized to the CCDP versus their current tool for completing IDPs (i.e., traditional IDP). The rationale for a cluster-randomized design versus an individual randomized design was twofold: (1) a cluster-randomized design minimizes contamination within sites (e.g., if participants were individually randomized, some mentors would have mentees using different IDP platforms, which may influence the behavior of both mentors and mentees) and (2) it is more practical for mentors, administrators, and program leadership to have all trainees and scholars use the same IDP platform. Relatedly, this also provides a more realistic assessment of this intervention as it would be implemented in the real world since a program will likely request that all its trainees and scholars use the same platform.

### Study settings and eligibility criteria

**Figure 1.**
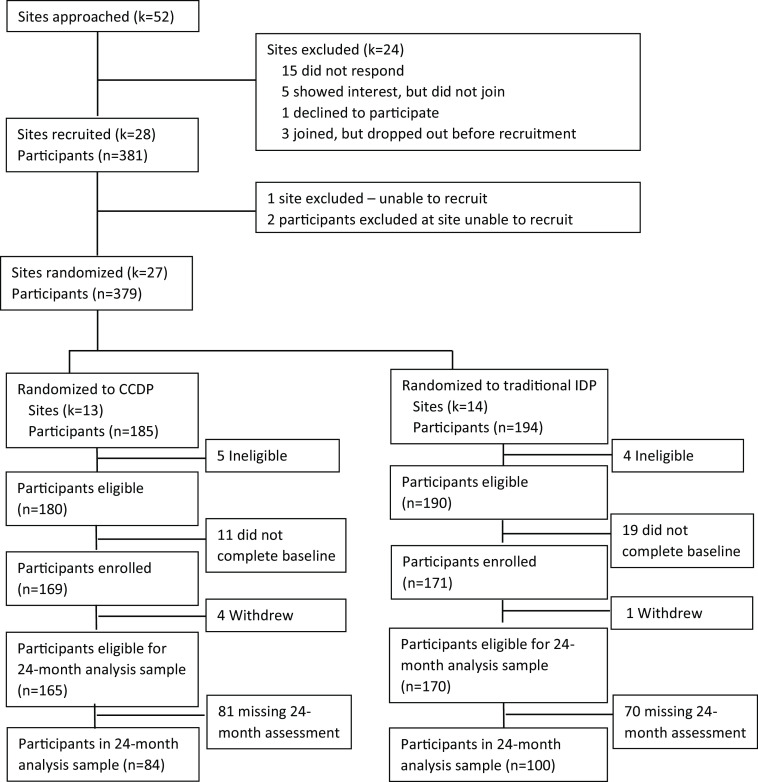
Clinical and Translational Science Award site and participant flow diagram for the customized career development platform trial.

We partnered with CTSA institutions to deliver the intervention. We had originally planned to enroll trainees and scholars from 24 participating CTSA hubs (12 sites randomized to each intervention arm); due to interest from other sites and concerns about achieving the overall target sample size, we decided to approach more sites than was needed for the sample size on which we were powered. Fifty-two CTSA hubs were approached to participate (see Consort Diagram, Fig. 1). We enrolled 27 sites and 340 participants (a complete list of the participating institutions is shown in Supplemental Table 1).

**Table 1.** Baseline characteristics of enrolled participants

	Total (*n* = 340)	CCDP (*n* = 169)	Traditional IDP (*n* = 171)	
	n (%)^a^	n (%)	n (%)	*P*-value
Age, mean (SD)	34 (6)	34 (7)	34 (6)	0.32
Range	21-58	21-54	23-58	
Gender				0.70
Male	132 (39.4)	68 (40.7)	64 (38.1)	
Female	202 (60.3)	99 (59.3)	103 (61.3)	
Non-binary	1 (0.3)	0 (0.0)	1 (0.6)	
Race				0.92
American Indian or Alaskan Native	2 (0.6)	1 (0.6)	1 (0.6)	
Asian	55 (17.2)	28 (17.8)	27 (16.6)	
Black or African American	33 (10.3)	16 (10.2)	17 (10.4)	
Caucasian	215 (67.2)	103 (65.6)	112 (68.7)	
Multi-racial	15 (4.7)	9 (5.7)	6 (3.7)	
Ethnicity				0.54
Hispanic or Latino	29 (9.1)	13 (8.1)	16 (10.1)	
Not Hispanic or Latino	291 (90.9)	148 (91.9)	143 (89.9)	
Disadvantaged background				0.36
Yes	71 (21.3)	32 (19.3)	39 (23.4)	
No	262 (78.7)	134 (80.7)	128 (76.7)	
Years in research, mean (SD)	8 (5)	8 (5)	8 (5)	0.89
Range	1-23	1-23	1-23	
Terminal degree				0.90
MD	120 (35.3)	61 (36.1)	59 (34.5)	
PhD	159 (46.8)	80 (47.3)	79 (46.2)	
MD/PhD	41 (12.1)	19 (11.2)	22 (12.9)	
PharmD	5 (1.5)	3 (1.8)	2 (1.2)	
Other^b^	15 (4.4)	6 (3.6)	9 (5.3)	
Career stage				0.23
Predoctoral fellow	25 (7.4)	10 (5.9)	15 (8.8)	
Postdoctoral fellow	137 (40.3)	63 (37.3)	74 (43.3)	
Faculty	178 (52.4)	96 (56.8)	82 (48.0)	
Peer-reviewed publications, mean (SD)	12.0 (12.9)	12.2 (12.3)	11.9 (13.4)	0.75
Range	1-91	1-62	1-91	
Have submitted a grant application				0.56
Yes	253 (79.8)	129 (81.1)	124 (78.5)	
No	64 (20.2)	30 (18.9)	34 (21.5)	
Grant applications submitted, mean (SD)	2.2 (2.1)	2.2 (2.1)	2.1 (2.0)	0.58
Range	0-10	0-10	0-10	
Job satisfaction score, mean (SD)	4.0 (0.5)	4.0 (0.5)	4.0 (0.6)	0.81
Range	2.1-5.0	2.6-5.0	2.1-5.0	
Career satisfaction score, mean (SD)	3.6 (0.7)	3.6 (0.7)	3.5 (0.7)	0.11
Range	1.6-5.0	1.6-5.0	1.6-5.0	

CCDP = customized career development plan; IDP = individual development plan; SD = standard deviation.

Missing baseline data by variable: age (*n* = 16), gender (*n* = 5), race (*n* = 16), ethnicity (*n* = 20), disadvantaged background (*n* = 7), and years in research (*n* = 95).

a Unless otherwise specified.

b Other highest degrees include DO, DPT, DVM, DVM/Ph.D., MD/MS, ND, PharmD/Ph.D., and VMD/Ph.D.

For the participants’ eligibility criteria, we intended to include any KL2 scholar or TL1 trainee. Prior to the initiation of recruitment, we broadened our eligibility criteria to any NIH-funded scholar or trainee at participating CTSA institutions who are required to complete an IDP, which allowed us to accommodate those sites that had other K scholars or T32 trainees. This expansion was agreed upon by the investigative team in response to feedback from CTSA sites that it was difficult for them to parse out just the KL2 or TL1 as the larger group interacted as a whole. This also helped minimize contamination.

### Study interventions

Participants at CTSA hubs assigned to the intervention group were instructed to use the CCDP for their IDP. We defined a “usage event” as any log on to the CCDP platform, regardless of length of session. Participants at CTSA hubs assigned to the control group were not given any instructions for their IDP outside those routinely given by their institution (i.e., a “usual practice” control condition).

The CCDP links the 14 CTSA clinical and translational science competencies with goals (Supplemental Figure 2) [5]. Initially, trainees and scholars completed a self-assessment of their proficiency with each competency on a scale of 1 to 4 (1= no exposure, 2 = developing competence, 3 = competent, and 4 = advanced). Scholars and trainees are prompted to re-assess their competencies within the CCDP annually. The platform tracks competencies they are working on and have achieved.

Key features of the CCDP include:

1. The CCDP is competency-driven in that trainees and scholars self-assess their current competency state based on the 14 established CTSA clinical and translational science competencies [5]. The platform displays the competency level of accomplishment so that mentors and program administrators can monitor competency attainment by individual scholar and trainee. The platform is flexible in that program administrators can select any of the CTSA competencies and add their own competencies for their program.
2. The CCDP allows scholars and trainees to specify their goals, link CTSA clinical and translational science competencies to each goal, set milestones, and generate timelines. This enables them to map out their career plans, track progress, and communicate with their mentoring teams to ultimately achieve their goals.
3. The CCDP automatically builds a Gantt Chart project schedule that trainees and scholars can use when meeting with their mentors. This helps facilitate mentoring meetings and keep the team focused on milestones and timelines.
4. The CCDP is used interactively with mentors. Once the CCDP is completed, the mentors receive an automatic email with a link so that they can review the CCDP. Trainees and scholars can also request a meeting to discuss their development plan with their mentors directly through the CCDP.
5. The online format enables real-time accessibility for the trainees and scholars to modify the CCDP as plans change. It facilitates communication among the mentor team as mentors are notified when the plan has changed. The mentors can access the trainees’ and scholars’ plan and comment on goals, milestones, or timelines. Its online format makes the CCDP a dynamic tool.
6. The CCDP is a two-way interactive platform. If trainees and scholars have not accessed their CCDP for 6 months, they are automatically prompted to visit their CCDP and make updates as appropriate.

### Study Outcomes

Participants completed study surveys and submitted Curriculum Vitas (CVs) in June–October 2020 and again, 24 months later in June–October 2022. We originally proposed to use the number of goals achieved as the primary outcome, intending to count the number of goals achieved from the CCDP for the intervention group and abstract them from the paper IDP for the control group. However, it quickly became apparent that there was considerable variability in the control group IDPs, leading to concern that we would not get consistent data on goals achieved from the control group participants. Moreover, we were not able to get the goals for control institutions that used myIDP as this is online and only permits the trainee or scholar to access their IDP. We, therefore, changed our primary outcome to the number of published manuscripts in the 24 months after baseline assessment. We abstracted the number of published manuscripts from participants' CV’s. We selected the number of published manuscripts as the primary outcome because the CCDP is a tool that enables scholars to strategically plan their careers and set milestones; the number of published manuscripts is a concrete objective measure of research output.

Secondary outcomes included the self-reported number of internal and external grant proposals submitted, the number of grants funded abstracted from CVs, job and career satisfaction, IDP use (usage events), and increased communication with mentors, which we measured using a follow-up survey (survey available upon request). We hypothesized that scholars who used the online CCDP would report higher number of grant proposals submitted, higher number of grants funded, higher career satisfaction, and improved communication with their mentors.

As a secondary analysis, we assessed and compared the level of proficiency for the 14 CTSA competencies in the CCDP arm at two time points (i.e., the first and second competency assessments; Supplemental Figure 3). This analysis only includes participants from the CCDP arm of the trial because only participants in this arm of the trial completed the competency assessments. We hypothesized that proficiency level would improve between the first and second competency assessments.

### Statistical Analysis

We prepared our original power analysis using the number of achieved goals set at baseline. Using data from our trainees and scholars at the University of Pittsburgh, we assumed a mean number of 10 goals achieved per scholar and a standard deviation of 6 in the traditional IDP group. Power was calculated with 24 sites and 12 scholars and trainees per site (totaling 288 scholars in the analysis), allowing for ICC = 0.05, we would have 79.3% power to detect a 25% increase in the number of goals achieved with online CCDP versus paper IDP. However, we had 27 sites who participated with 340 scholars and trainees.

Since we changed our outcome to the number of publications once the study was underway, we did not recalculate our power analysis because randomization and recruitment were completed.

Sites were randomly assigned to the online CCDP or traditional IDP control with fixed block sizes of four to ensure approximately equal allocation to the two treatment arms. No stratification was performed.

We compared participants who were included in versus excluded from the 24-month analysis using descriptive statistics (i.e., chi-square test for categorical variables and Wilcoxon rank-sum test for continuous variables). To compare the number of published manuscripts during the first 24 months after the baseline visit between the two intervention groups, we used generalized linear mixed models with a log-link function, modeling the number of manuscripts published as count data. The model included fixed effects for the intervention group and prespecified factors, such as type of terminal degree (i.e., MD, PhD, MD/PhD, PharmD, or other) and career level (i.e., predoctoral fellow, postdoctoral fellow, or early-career faculty). We also included a random effect for the CTSA hub to account for the cluster-randomized design. The same analytic method was used for secondary end points, such as the number of grant proposals submitted and the number of grants funded. We used linear mixed models with fixed effects for the type of terminal degree and career level and random effects for CTSA hub to compare job and career satisfaction scores between intervention groups. We used mixed-effects ordinal logistic regression models with the same fixed and random effects to compare IDP use and communication with mentor(s) between intervention groups.

### Competency Assessment

Descriptive statistics were used to characterize the population in the CCDP arm who completed the first and second competency assessments and summarize the change in level of proficiency of each competency between the two time points. We tested whether proficiency level changed from the first to second assessment using the Wilcoxon signed-rank test. We also report the percent of participants with improvement in their proficiency level between the first and second competency assessment (i.e., had no exposure at first assessment and developing competence or competent/advanced at second assessment; or was developing competence at the first assessment and competent/advanced at the second assessment).

## Results

Three-hundred and forty participants completed the baseline and were fully enrolled in the study. The median number of participating trainees and scholars per institution was 12 (minimum 7; maximum 28). Baseline demographics of the study participants are shown in Table 1. The mean age of participants was 34 years (range 21–58 years). Participants were mostly female (60.3%) and predominantly Caucasian (67.2%). The most common terminal degree was a PhD (46.8%), followed by MD (35.3%) and MD/PhD (12.1%). Those excluded versus those included in the 24-month analyses had fewer baseline publications, were more likely to be MDs, and had higher pre-intervention job satisfaction (Supplemental Table 2).

**Table 2.** Comparison of the number of publications, grant applications submitted, grant applications funded, and job and career satisfaction scores between CCDP and traditional IDP groups at 24 months post-intervention

	CCDP (*n* = 84) Mean (SD)	Traditional IDP (*n* = 100)Mean (SD)	Adjusted mean difference (95% CI)	*P*-value
Number of publications	9.4 (9.6)	8.6 (9.1)	0.08 (−0.42 − 0.57)	0.76
Number of grant applications submitted since baseline	4.1 (3.5)	4.4 (3.4)	−0.16 (−0.49 − 0.16)	0.32
Pilot award applications submitted	2.3 (1.2)	2.3 (1.7)	0.003 (−0.38 − 0.39)	0.99
K award or similar career development award applications submitted	2.1 (1.6)	1.7 (1.0)	0.18 (−0.24 − 0.60)	0.38
R01 or equivalent award applications submitted	2.4 (1.8)	2.5 (1.4)	−0.03 (−0.43 − 0.37)	0.87
R03 or R21 award applications submitted	2.1 (1.3)	1.8 (0.8)	0.15 (−0.56 − 0.86)	0.63
Other applications submitted	2.0 (1.3)	2.4 (1.8)	−0.19 (−0.57 − 0.19)	0.32
Number of grant applications funded since baseline	1.3 (1.6)	2.0 (2.5)	−0.23 (−0.77 − 0.31)	0.40
Job satisfaction score^b^	3.6 (0.7)	3.7 (0.6)	−0.09 (−0.31 − 0.12)	0.38
Career satisfaction score^c^	3.7 (0.6)	3.6 (0.7)	0.10 (−0.12 − 0.32)	0.38

CCDP = customized career development plan; CI = confidence interval; IDP = individual development plan; SD = standard deviation.

a Adjusted mean differences for count data between randomized IDP assignment at 24-month assessment were estimated with generalized linear mixed models with log-link function, including fixed effects for career level and type of highest degree, and random effects for Clinical and Translational Science Award hub. Results presented as adjusted mean difference (95% CI) for the CCDP versus traditional IDP group. Unadjusted mean (SD) are also reported for ease of interpretation. Linear mixed models for continuous outcomes (i.e., job satisfaction score and career satisfaction score) with fixed effects for type of terminal degree, and career level and random effects for Clinical and Translational Science Award hub.

b Job satisfaction score range: 1.0–5.0 with higher scores indicating greater job satisfaction.

c Career satisfaction score range: 1.0–5.0, with higher scores indicating greater career satisfaction.

At 24 months post-intervention, the CCDP versus traditional IDP groups were similar in the mean number of publications (9.4 vs 8.6; *p* = 0.76), number of grants submitted (4.1 vs 4.4; *p* = 0.32), number of grants funded (1.3 vs 2.0; *p* = 0.40), job satisfaction score (3.6 vs 3.7; *p* = 0.38), and career satisfaction (3.7 vs 3.6; *p* = 0.38; Table 2). There was no difference between the CCDP and traditional IDP groups in the perceived help that their IDP provided (Table 3). The CCDP group had lower odds of the IDP helping facilitate meetings with mentors (OR = 0.56, *p* = 0.04; Table 3) and higher odds of discussing communication skills (OR = 2.08; *p* = 0.02), leadership skills (OR = 2.62; *p* = 0.003), and broadening their network (OR = 2.31; *p* = 0.01) to a greater extent than the traditional IDP group (Table 4).

**Table 3.** Comparison of individual development plan use between CCDP and traditional idp groups at 24 months post–intervention

	CCDP (*n* = 83) n(%)	Traditional IDP (*n* = 100)n(%)	OR^a^ (95% CI)	*P*-value
Frequency of IDP revisions			0.88 (0.45–1.73)	0.72
Never	17 (20.5)	18 (18.0)		
Once	43 (51.8)	52 (52.0)		
Every 6 months	18 (21.7)	22 (22.0)		
At least quarterly	5 (6.0)	8 (8.0)		
*IDP helped me*				
Make plans to meet professional goals			0.68 (0.38–1.21)	0.19
1 – Not at all	27 (32.5)	21 (21.4)		
2	15 (18.1)	22 (22.5)		
3	21 (25.3)	24 (24.5)		
4 or 5 – Large extent	20 (24.1)	31 (31.6)		
Track exposure to clinical and translational research competencies			1.04 (0.59–1.84)	0.88
1 – Not at all	27 (32.9)	26 (26.8)		
2	11 (13.4)	22 (22.7)		
3	25 (30.5)	26 (26.8)		
4 or 5 – Large extent	19 (23.2)	23 (23.8)		
Facilitate meetings with mentors			0.56 (0.32–0.97)	0.04
1– Not at all	36 (43.9)	30 (30.9)		
2	14 (17.1)	18 (18.6)		
3	17 (20.7)	20 (20.6)		
4 or 5 – Large extent	15 (18.3)	29 (30.0)		

CCDP = customized career development plan; CI = confidence interval; IDP = individual development plan; OR = odds ratio.

a Mixed-effects ordinal logistic regression models were used to compare the IDP use between the CCDP and traditional IDP groups, including fixed effects for type of terminal degree and career level and random effects for Clinical and Translational Science Award hub.

**Table 4.** Comparison of mentoring between CCDP and traditional IDP groups at 24 months post–intervention

	CCDP (*n* = 70) *n* (%)	Traditional IDP (*n* = 90) *n* (%)	OR^a^ (95% CI)	*P*-value
Monthly number of regularly scheduled or impromptu meetings with mentor			1.75 (0.75–4.08)	0.20
≤1	15 (21.4)	25 (27.8)		
2–3	19 (27.1)	28 (31.1)		
4–5	22 (31.4)	24 (26.7)		
6+	14 (20.0)	13 (14.4)		
Monthly number of emails, phone, text messaging, or social network communication with mentor			0.95 (0.48–1.87)	0.88
≤1	12 (17.4)	16 (18.0)		
2–3	19 (27.5)	20 (22.5)		
4–5	11 (15.9)	15 (16.9)		
6+	27 (39.1)	38 (42.7)		
Amount of mentoring received at your institution to help you:				
Formulate your career goal			1.70 (0.92–3.12)	0.09
None to a little	12 (17.2)	23 (24.7)		
A moderate amount	30 (42.9)	41 (46.1)		
A lot	28 (40.0)	26 (29.2)		
Plan how to achieve your career goals			1.66 (0.78–3.52)	0.19
None to a little	14 (20.0)	23 (25.9)		
A moderate amount	28 (40.0)	40 (44.9)		
A lot	28 (40.0)	26 (29.2)		
Learn the skills needed to succeed in your career goals			1.33 (0.65–2.71)	0.43
None to a little	14 (20.0)	13 (14.6)		
A moderate amount	29 (41.4)	53 (59.6)		
A lot	27 (38.6)	23 (25.8)		
Find the resources you need			1.32 (0.67–2.62)	0.42
None to a little	12 (17.2)	20 (22.5)		
A moderate amount	35 (50.0)	44 (49.4)		
A lot	23 (32.9)	25 (28.1)		
Have a sponsor/champion to advance your career or work			1.34 (0.73–2.46)	0.34
None	14 (20.3)	21 (23.6)		
A moderate amount	25 (36.2)	36 (40.5)		
A lot	30 (43.5)	32 (36.0)		
Plan to achieve your personal goals			1.36 (0.68–2.74)	0.38
None to a little	24 (35.3)	37 (42.0)		
A moderate amount	28 (41.2)	31 (35.2)		
A lot	16 (23.5)	20 (22.7)		
Extent you addressed each of the following with your mentor(s)				
Research skills and scientific techniques, concepts, and approaches			1.51 (0.56–4.02)	0.41
1 or 2 – Not at all	5 (7.1)	5 (5.7)		
3	9 (12.9)	18 (20.2)		
4 or 5 – Large extent	56 (80.0)	66 (74.2)		
Communication skills			2.08 (1.12–3.83)	0.02
1 or 2 – Not at all	13 (18.6)	29 (32.5)		
3	19 (27.1)	27 (30.3)		
4 or 5 – Large extent	38 (54.3)	33 (37.1)		
Leadership skills			2.62 (1.40–4.87)	0.003
1 or 2 – Not at all	15 (21.5)	27 (30.4)		
3	14 (20.0)	34 (38.2)		
4 or 5 – Large extent	41 (58.6)	28 (31.5)		
Broadening your network			2.31 (1.20–4.45)	0.01
1 or 2 – Not at all	11 (15.8)	23 (26.2)		
3	14 (20.0)	25 (28.4)		
4 or 5 – Large extent	45 (64.3)	40 (45.5)		
Career planning and developing independence			1.29 (0.67–2.47)	0.44
1 or 2 – Not at all	13 (18.6)	13 (14.6)		
3	12 (17.1)	25 (28.1)		
4 or 5 – Large extent	45 (64.3)	51 (57.4)		
Educational choices and strategies			1.29 (0.69–2.38)	0.42
1 or 2 – Not at all	26 (37.7)	30 (33.8)		
3	10 (14.5)	25 (28.1)		
4 or 5 – Large extent	33 (47.8)	34 (38.2)		
Grant writing and/or seeking funding for research			1.38 (0.63–3.03)	0.42
1 or 2 – Not at all	7 (10.0)	9 (10.1)		
3	9 (12.9)	16 (18.0)		
4 or 5 – Large extent	54 (77.2)	64 (71.9)		
Life events			0.95 (0.52–1.74)	0.88
1 or 2 – Not at all	27 (39.1)	31 (35.2)		
3	20 (29.0)	28 (31.8)		
4 or 5 – Large extent	22 (31.9)	29 (33.0)		
Work–life integration challenges			0.91 (0.50–1.67)	0.76
1 or 2 – Not at all	33 (47.8)	35 (39.4)		
3	16 (23.2)	30 (33.7)		
4 or 5 – Large extent	20 (29.0)	24 (27.0)		
Diversity and inclusion			0.91 (0.49–1.71)	0.78
1 or 2 – Not at all	37 (53.6)	43 (48.3)		
3	17 (24.6)	23 (25.8)		
4 or 5 – Large extent	15 (21.7)	23 (25.8)		
Self-efficacy/confidence			0.84 (0.41–1.74)	0.64
1 or 2 – Not at all	32 (46.3)	35 (39.3)		
3	21 (30.4)	28 (31.5)		
4 or 5 – Large extent	16 (23.2)	26 (29.2)		
Motivation			1.29 (0.68–2.44)	0.43
1 or 2 – Not at all	28 (40.6)	39 (43.8)		
3	21 (30.4)	25 (28.1)		
4 or 5 – Large extent	20 (29.0)	25 (28.1)		
Your IDP			1.03 (0.54–1.95)	0.94
1 or 2 – Not at all	41 (58.6)	52 (59.7)		
3	16 (22.9)	15 (17.2)		
4 or 5 – Large extent	13 (18.6)	20 (23.0)		

CCDP = customized career development plan; CI = confidence interval; IDP = individual development plan; OR = odds ratio.

a Mixed-effects ordinal logistic regression models were used to compare the IDP use between the CCDP and traditional IDP groups, including fixed effects for type of terminal degree and career level and random effects for Clinical and Translational Science Award hub.

### Competency Assessment

One hundred and twenty-six out of 169 participants (75%) in the CCDP arm of the trial completed at least one competency assessment. Supplemental Table 3 shows participant characteristics for those who completed competency assessments. Forty-eight percent (*n* = 60) of participants who completed a first competency assessment also completed a second assessment. The median length of time between completion of the first and second competency assessment was 13 months (25^th^–75^th^ percentile: 7–16 months).

Among participants in the CCDP arm who completed the first and second competency assessments, there were significant proficiency-level improvements in *biomedical informatics*, *clinical and translational research questions*, *cross disciplinary training*, *leadership*, *literature critique*, *research implementation, statistical approaches*, *study design,* and *translational teamwork* competencies (p for all<0.05). There were no significant improvements in the self-assessed proficiency level for *clinical research interactions* (*p* = 0.31), *community engagement* (*p* = 0.62), *cultural diversity* (*p* = 0.17), *scientific communication* (*p* = 0.06), and *sources of error* (*p* = 0.07; Table 5) competencies. Among those who could improve their proficiency level between the first and second competency assessments, a range of improvement was observed (30% for *leadership* competency to 61% for *literature critique* competency; Table 6).

**Table 5.** Comparison of level of Clinical and Translational Science Award competency at first and second assessment

	1^st^ assessment		2^nd^ assessment		
	*n* = 126	(%)	*n* = 60	(%)	*P*-value
Biomedical informatics					0.02
No exposure	27	(21.4)	8	(13.3)	
Developing competence	74	(58.7)	31	(51.7)	
Competent/advanced	25	(19.8)	21	(35.0)	
Clinical research interactions					0.31
No exposure	5	(4.2)	1	(1.7)	
Developing competence	50	(42.0)	22	(37.3)	
Competent/advanced	64	(53.8)	36	(61.0)	
Clinical and translational research questions					0.01
No exposure	5	(4.0)	0	(0.0)	
Developing competence	50	(39.7)	15	(25.0)	
Competent/advanced	71	(56.4)	45	(75.0)	
Community engagement					0.62
No exposure	25	(19.8)	10	(16.7)	
Developing competence	64	(50.8)	31	(51.7)	
Competent/advanced	37	(29.4)	19	(31.7)	
Cross disciplinary training					< 0.001
No exposure	10	(8.4)	0	(0.0)	
Developing competence	66	(55.5)	21	(35.6)	
Competent/advanced	43	(36.1)	38	(64.4)	
Cultural diversity					0.17
No exposure	11	(8.7)	2	(3.3)	
Developing competence	57	(45.2)	25	(41.7)	
Competent/advanced	58	(46.0)	33	(55.0)	
Leadership					0.04
No exposure	3	(2.4)	0	(0.0)	
Developing competence	71	(57.7)	27	(45.0)	
Competent/advanced	49	(39.8)	33	(55.0)	
Literature critique					0.02
No exposure	1	(0.8)	0	(0.0)	
Developing competence	46	(36.5)	12	(20.0)	
Competent/advanced	79	(62.7)	48	(80.0)	
Research implementation					0.04
No exposure	4	(3.4)	0	(0.0)	
Developing competence	57	(47.9)	21	(35.6)	
Competent/advanced	58	(48.7)	38	(64.4)	
Scientific communication					0.06
No exposure	2	(1.6)	0	(0.0)	
Developing competence	60	(47.6)	21	(35.0)	
Competent/advanced	64	(50.8)	39	(65.0)	
Sources of error					0.07
No exposure	2	(1.7)	1	(1.7)	
Developing competence	66	(55.5)	24	(40.7)	
Competent/advanced	51	(42.9)	34	(57.6)	
Statistical approaches					0.02
No exposure	6	(4.8)	0	(0.0)	
Developing competence	66	(52.4)	24	(40.0)	
Competent/advanced	54	(42.9)	36	(60.0)	
Study design					0.01
No exposure	3	(2.4)	0	(0.0)	
Developing competence	57	(45.2)	17	(28.3)	
Competent/advanced	66	(52.4)	43	(71.7)	
Translational teamwork					0.046
No exposure	8	(6.7)	0	(0.0)	
Developing competence	64	(53.8)	28	(47.5)	
Competent/advanced	47	(39.5)	31	(52.5)	

**Table 6.** Improvement in competencies between first and second assessment among those who could improve

	*n* of those who could improve	Improved	
Competencies		*n*	(%)
Biomedical informatics	51	18	(35.3)
Clinical research interactions	38	17	(44.7)
Clinical and translational research questions	34	20	(58.8)
Community engagement	53	19	(35.9)
Cross-disciplinary training	41	23	(56.1)
Cultural diversity	40	18	(45.0)
Leadership	39	12	(30.8)
Literature critique	31	19	(61.3)
Research implementation	37	17	(46.0)
Scientific communication	40	20	(50.0)
Sources of error	38	13	(34.2)
Statistical approaches	37	15	(40.5)
Study design	31	15	(48.4)
Translational teamwork	43	18	(41.9)

## Discussion

We conducted a clustered-randomized controlled trial comparing the online CCDP versus traditional IDP use among 340 geographically diverse clinical and translational scholars and trainees. We hypothesized that 24-month post-intervention, scholars and trainees randomized to the CCDP arm would report higher number of published manuscripts compared to those assigned to their traditional IDP. We found no differences between the CCDP and traditional IDP arms for our primary outcome, the number of published manuscripts 24 months after baseline assessment.

Analysis of data for our secondary outcomes (self-reported number of grant proposals submitted, number of grants funded abstracted from CVs, scholars’ career satisfaction, IDP use, and communication with mentors) and competency assessments provided an important mix of results. For example, those on the CCDP arm showed statistically significant higher odds of discussing communication and leadership skills and broadening their network to a greater extent than the traditional IDP group. Similarly, when examining the CCDP group’s competencies, there were significant improvements in *cross-disciplinary training, leadership,* and *translational teamwork.* These similarities may be due to the CCDP arm focusing on tying competencies to the scholar’s goalsetting, thereby facilitating additional communication touch points with their mentors. As described by Abedin *et al*. [6] for early-career CTSA investigators, communication and interpersonal skills are core elements in building mentoring relationships and professional networks, which the CCDP appears to support. On the other hand, CCDP users reported lower odds of the CCDP helping facilitate meetings with mentors than traditional IDP users, results that seem somewhat contradictory. One possible explanation is that the CCDP allowed mentees and mentors to communicate within the online platform more efficiently and effectively. Since the CCDP online platform allows goal setting, competency monitoring, and goal achievement to be updated and shared automatically between mentors and mentees, it enables more frequent communication opportunities online. Unlike the traditional IDP group, those in the CCDP arm may not have required as many offline meetings. Although scholars were able to request a meeting through the CCDP platform, we speculate they did not use this feature because they still had to work with the mentor or mentor’s administrative assistant to do the actual scheduling. We did not survey mentors, so cannot speculate on the mentors’ level of engagement with the mentee and the CCDP; it is possible that mentors did not follow up with their trainees, and it is also possible that the trainee did not share their CCDP with their mentor. Although we found statistically significant changes in reported levels of competency for most of the 14 competencies, a possible reason some did not change could be related to that participant’s research area, or if their program does not emphasize that competency. Differences between the two groups for other secondary outcomes were not educationally meaningful nor statistically significant.

Despite the advantages of the CCDP versus paper IDPs described above, the data from the participants in the CCDP arm did not reflect an appreciation for these advantages, nor did they reflect enthusiasm for use. However, as the literature describes, this aligns with the general lack of enthusiasm for IDPs. Our findings support the lack of appreciation for the benefits of IDPs found by Smyth *et al*. [4] and the lack of compliance with the requirement to use an IDP by K and T trainees described by Martina, Gabrilove, Luban, and Patino [3]. Less than half of participants in the CCDP arm completed more than one competency assessment, suggesting that the CCDP was not used as intended. Because of this perceived lack of enthusiasm, we speculate that elements of Rogers’ Diffusion of Innovation theory and his innovation-decision process may partly be at play [7]. This theory, a widely used theoretical framework in the area of technology diffusion and adoption, explains how an idea or use of an innovative product spreads through a population over time. Rogers describes a phenomenon called “disenchantment discontinuance.” Disenchantment discontinuance occurs when the participant stops using the innovation because they: (1) are not satisfied with its performance; (2) feel it does not meet their needs; or (3) do not perceive a relative advantage between the innovation and “usual use [8].” It would be beneficial to interview scholars to gain an understanding of their barriers to use and whether that included the design of the tool.

Another possible reason for the lack of differences in publications is the fact that the study started during the pandemic. We know that women were publishing less than men [9] and most of the participants were women (60%). This lower number of publications may have impacted the effect size so that we were unable to see significant difference between groups.

Follow-up research, including focus groups or qualitative interviews, may shed light on this speculation. It simply may be that our participants fit the characteristics of Rogers’ “late majority” adopters – those skeptical of change, who will only adopt an innovation after the majority has tried it [7].

Our study has several strengths and limitations. Our study is novel because it tested the effectiveness of a new electronic IDP tailored to a diverse group of clinical and translational research pre- and postdoctoral trainees, early-career faculty scholars, and their mentors. While the CCDP was designed for CTSA hubs, it has the potential for broader appeal. Unlike other IDPs, any research training program could adopt the CCDP to plan, track, and evaluate their trainees' research and career development success. Though it was built using the NIH CTSA clinical and translational research competencies, program administrators are encouraged to customize the CCDP to their needs by selecting which competencies are relevant for individual programs and deleting and adding competencies beyond the original 14 clinical and translational research competencies. Furthermore, this was a large pragmatic trial with geographically diverse CTSA hubs. The limitations of this study included the high loss to follow-up experienced at 24 months post-intervention, the low uptake of the competency assessments in the CCDP arm, the heterogeneity in the traditional IDP tools, the variability in the traditional IDPs submitted from a single site (i.e., no standard template), the reliance on self-report data, and the unforeseen impact of COVID-19 during our study period on research, communication, and mentoring relationships. We also did not collect information on how the CCDP was being used and suspect that it was not used to the fullest extent by many scholars and mentors. Future research could explore use, usability, and satisfaction by the scholars as well as the mentors.

In conclusion, the CCDP offers CTSA hubs an innovative option to traditional IDP tools. The CCDP has the advantages of being online, interactive, accessible in real time, and tailored to CTSA competencies. While our study did not show a difference in research productivity (e.g., publications or grants) or career satisfaction between the CCDP and traditional IDP groups, we did find the CCDP group were more likely to address key topics (e.g., communication skills, leadership skills, and networking) with their mentors. The CCDP users also reported increased proficiency in 9 of the 14 CTSA established competencies. Future studies are needed to elucidate why the CCDP users did not fully appreciate or adopt the functionality of the platform as intended and how to build strategies to improve implementation and longitudinal engagement.

## Supporting information

Rubio et al. supplementary material Rubio et al. supplementary material

## Supplementary material

The supplementary material for this article can be found at https://doi.org/10.1017/cts.2023.687.
