# Supplementary material for: Customized Career Development Platform (CCDP) for clinical and translational researchers: A pragmatic cluster-randomized controlled trial
Source: J Clin Transl Sci. 2023 Nov 22;7(1):e259. doi: 10.1017/cts.2023.687 (PMC10790233; doi:10.1017/cts.2023.687)
Supplement: Rubio et al. supplementary material [file S2059866123006878sup001.pdf]

Supplemental Figure 1. Screenshot of CCDP Gantt Chart. Scholars set goals by category and link them with competencies. Mentors can view and provide comments or schedule meetings with Scholars.

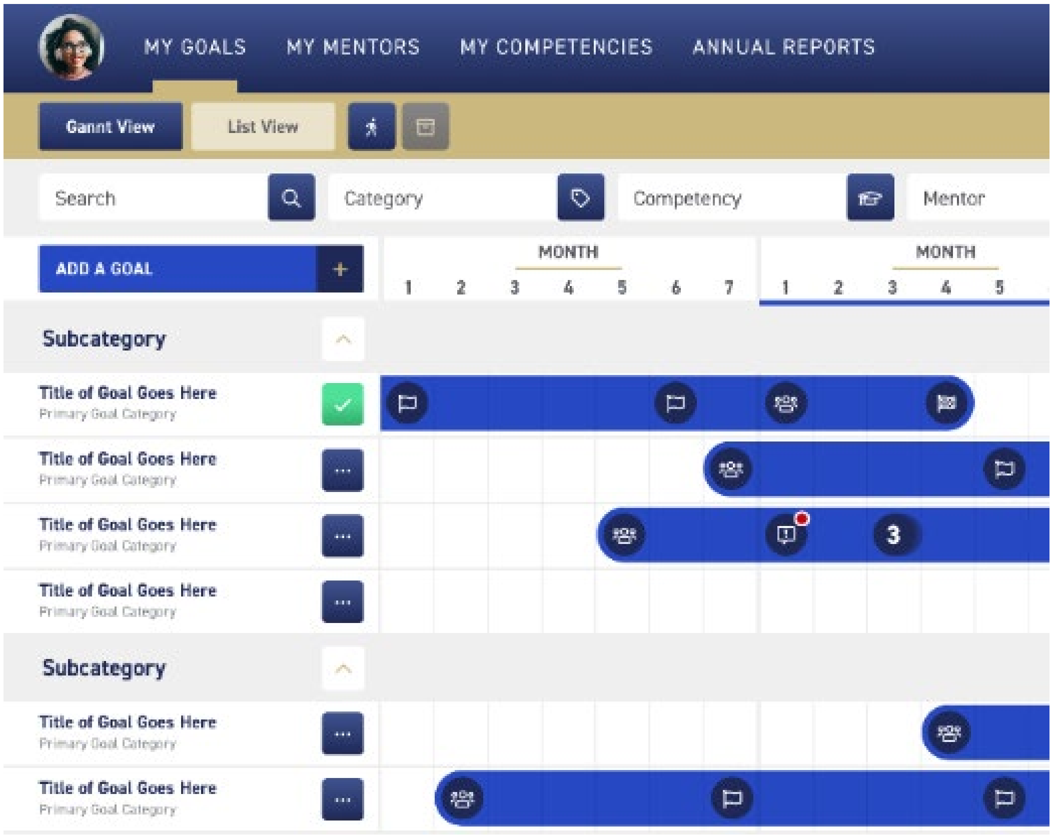

Supplemental Figure 2. Display of Competencies in the CCDP

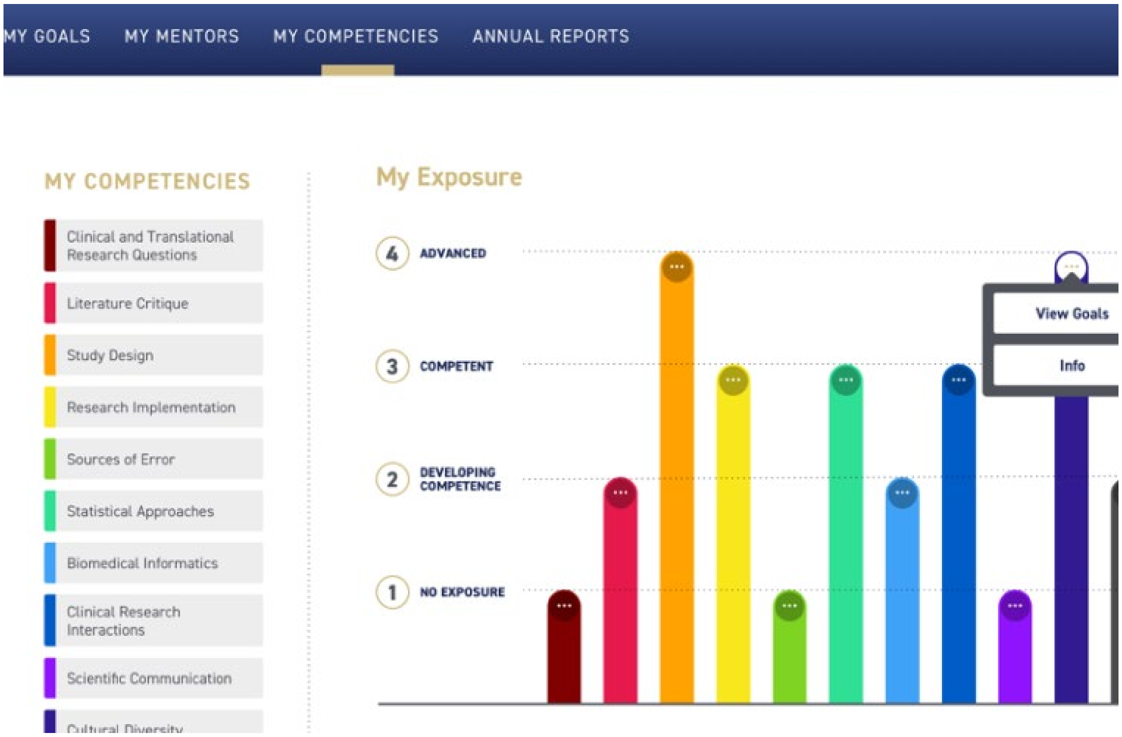

Supplemental Figure 3. Participant Flow Diagram for the Customized Career Development Platform (CCDP) Trial

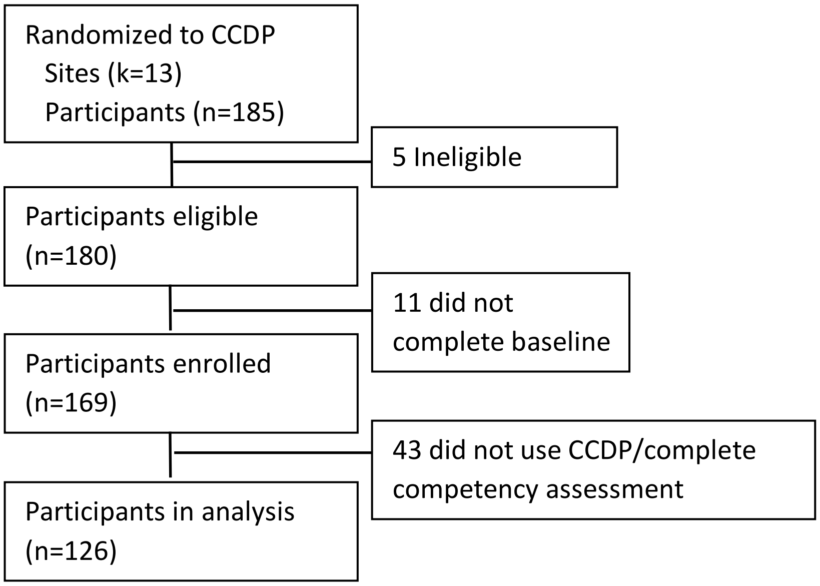

Supplemental Table 1. Complete List of Participating Clinical and Translational Science Award Hubs

Clinical and Translational Science Institute at Children's National: A Partnership with The George Washington University
Colorado Clinical and Translational Sciences Institute
Columbia Irving Institute for Clinical and Translational Research
ConduITS Institute for Translational Sciences at the Icahn School of Medicine at Mount Sinai
Duke Clinical and Translational Science Institute
Georgetown-Howard Universities Center for Clinical and Translational Science
Indiana Clinical and Translational Sciences Institute
Johns Hopkins Institute for Clinical & Translational Research
Michigan Institute for Clinical and Health Research
North Carolina Translational and Clinical Sciences Institute (NC TraCS)
Oregon Clinical and Translational Research Institute
Penn State Clinical and Translational Science Institute
Rockefeller University Center for Clinical and Translational Sciences
Scripps Research Translational Institute
Southern California Clinical and Translational Science Institute
UC Davis Health Clinical and Translational Science Center
UC San Diego Altman Clinical and Translational Research Institute
UCLA Clinical and Translational Science Institute
UCSF Clinical and Translational Science Institute
University of Alabama at Birmingham Center for Clinical and Translational Science
University of Florida Clinical and Translational Science Institute
University of Kentucky Center for Clinical and Translational Science
University of Pennsylvania Institute for Translational Medicine and Therapeutics
University of Rochester Clinical and Translational Science Institute
Utah Clinical and Translational Science Institute
UW Institute for Clinical and Translational Research
Washington University Institute of Clinical and Translational Sciences

Supplemental Table 2. Comparison of those with and without 24-month data

	Included in 24-month analysis (n=184) n (%)[a]	Excluded from 24-month analysis (n=156) n (%)	P-value
Age, mean (SD)	34 (6)	34 (6)	0.45
Gender			0.81
Male	69 (38.1)	63 (40.9)	
Female	111 (61.3)	91 (59.1)	
Non-Binary	1 (0.6)	0 (0.0)	
Race[1]			0.11
American Indian or Alaskan Native	1 (0.6)	1 (0.7)	
Asian	24 (13.8)	31 (21.2)	
Black or African American	14 (8.1)	19 (13.0)	
Native Hawaiian or Pacific Islander	0 (0.0)	0 (0.0)	
Caucasian	124 (71.3)	91 (62.3)	
Multi-Racial	11 (6.3)	4 (2.7)	
Ethnicity			0.68
Hispanic or Latino	15 (8.5)	14 (9.8)	
Not Hispanic or Latino	162 (91.5)	129 (90.2)	
Disadvantaged Background			0.99
Yes	38 (21.4)	33 (21.3)	
No	140 (78.7)	122 (78.7)	
Years in Research, mean (SD)	8 (5)	8 (5)	0.47
Range	1-23	1-23	
Degree			<0.01
MD	53 (28.8)	67 (43.0)	
PhD	101 (54.9)	58 (37.2)	
MD/PhD	17 (9.2)	24 (15.4)	
PharmD	5 (2.7)	0 (0.0)	
Other	8 (4.4)	7 (4.5)	
Career stage			0.36
Pre-doctoral fellow	11 (6.0)	14 (9.0)	
Post-doctoral fellow	71 (38.6)	66 (42.3)	
Faculty	102 (55.4)	76 (48.7)	
Peer-reviewed publications at baseline, mean (SD)	14.4 (15.2)	8.9 (7.9)	0.01
Range	1-91	1-36	
Grants submitted at baseline, mean (SD)	2.4 (2.2)	1.9 (1.8)	0.10
Range	0-10	0-10	
Job satisfaction score, mean (SD)	3.7 (0.7)	4.0 (0.5)	<0.01

Range	0.3-5.0	2.1-5.0	
Career satisfaction score, mean (SD)	3.6 (0.7)	3.6 (0.6)	0.27
Range	0.8-5.0	1.6-5.0	
Intervention group			0.10
Traditional IDP	100 (54.4)	71 (45.5)	
CCDP	84 (45.7)	85 (54.5)	

CCDP, customized career development plan; IDP, individual development plan; SD, standard deviation.

[a]Unless otherwise specified.

Supplemental Table 3. Characteristics of Participants Randomized to the Customized Career Development Platform Trial at First and Second Competency Assessment

	1st assessment		2nd assessment	
Characteristic	n=126	(%)	n=60	(%)
Age (median, 25th-75th percentile)	34	(27-38)	35	(32-39)
Gender				
Male	53	(42.7)	28	(48.3)
Female	71	(57.3)	30	(51.7)
Race				
White	73	(63.5)	39	(69.6)
Black	12	(10.4)	7	(12.5)
Asian	22	(19.1)	8	(14.3)
Multi-race	8	(7.0)	2	(3.6)
Hispanic/Latinx ethnicity				
Yes	11	(9.2)	3	(5.4)
No	108	(90.8)	53	(94.6)
Disadvantaged background				
Yes	21	(17.1)	11	(18.6)
No	102	(82.9)	48	(81.4)
Underrepresented in sciences				
Yes	35	(29.9)	15	(26.8)
No	82	(70.1)	41	(73.2)
Highest degree				
MD	48	(38.1)	18	(30.0)
PhD	60	(47.6)	30	(50.0)
Other	18	(14.3)	12	(20.0)
Career status				
Pre-doctoral fellow	9	(7.1)	2	(3.3)
Post-doctoral fellow	54	(42.9)	24	(40.0)
Faculty	63	(50.0)	34	(56.7)
